# Increased expression of urokinase plasminogen activator and its cognate receptor in human seminomas

**DOI:** 10.1186/1471-2407-10-151

**Published:** 2010-04-19

**Authors:** Salvatore Ulisse, Enke Baldini, Marcella Mottolese, Steno Sentinelli, Patrizia Gargiulo, Brancato Valentina, Salvatore Sorrenti, Anna Di Benedetto, Enrico De Antoni, Massimino D'Armiento

**Affiliations:** 1 Department of Experimental Medicine, "Sapienza" University of Rome, Italy; 2 Department of Pathology, Regina Elena Cancer Institute, Rome, Italy; 3 Department of Surgical Sciences, "Sapienza" University of Rome, Italy

## Abstract

**Background:**

The urokinase plasminogen activating system (uPAS) is implicated in neoplastic progression and high tissue levels of uPAS components correlate with a poor prognosis in different human cancers. Despite that, relative few studies are available on the expression and function of the uPAS components in human seminomas. In the present study we characterized the expression of the urokinase plasminogen activator (uPA), its cognate receptor (uPAR) and the uPA inhibitors PAI-1 and PAI-2 in normal human testis and seminomas.

**Methods:**

The expression of the above genes was evaluated by means of quantitative RT-PCR, western blot, zymographic analysis and immunohistochemistry.

**Results:**

Quantitative RT-PCR analysis of 14 seminomas demonstrated that uPA and uPAR mRNAs were, with respect to control tissues, increased in tumor tissues by 3.80 ± 0.74 (p < 0.01) and 6.25 ± 1.18 (p < 0.01) fold, respectively. On the other hand, PAI-1 mRNA level was unchanged (1.02 ± 0.24 fold), while that of PAI-2 was significantly reduced to 0.34 ± 0.18 (p < 0.01) fold. Western blot experiments performed with protein extracts of three seminomas and normal tissues from the same patients showed that uPA protein levels were low or undetectable in normal tissues and induced in tumor tissues. On the same samples, zymographic analysis demonstrated increased uPA activity in tumor tissue extracts. Western blot experiments showed that also the uPAR protein was increased in tumor tissues by 1.83 ± 0.15 fold (p < 0.01). The increased expression of uPA and uPAR was further confirmed by immunohistochemical staining performed in 10 seminomas and autologous uninvolved peritumoral tissues. Finally, variation in the mRNA level of PAI-1 significantly correlated with tumor size.

**Conclusions:**

We demonstrated the increased expression of uPA and uPAR in human seminomas with respect to normal testis tissues, which may be relevant in testicular cancer progression.

## Background

The term "germ cell tumors" refers to a heterogeneous group of neoplasms originating from cells belonging to the germ cell lineage [1-3]. They occur mainly in the gonad, but also in specific extragonadal sites along the migration route of primordial germ cells. In the human, testis germ cell tumors comprise three main entities characterized by different epidemiological, histological and clinical parameters. The first includes the teratomas-yolk sac tumors usually taking place during the first years of life; the second includes the testicular germ cell tumors (TGCT) and consists of seminoma and non-seminoma cancers taking place following puberty and during the adult life; the last is represented by the spermatocytic seminomas which become manifest in elderly men [2,3]. Although germ cell tumors are rare in the male population, accounting for less than 1% of all cancers, the TGCT is the most common malignancy in young adult caucasian males [3,4]. Overt TGCT is thought to generate from a precursor neoplastic lesion defined as intratubular germ cell neoplasia (IGCN) [3,5,6]. The malignant progression of the IGCN, characterized by extratubular invasion, is thought to be an active process requiring the breakdown of the extracellular matrix (ECM) and the basement membrane (BM) surrounding the seminiferous tubules [3].

The urokinase plasminogen activating system (uPAS) consists of the urokinase plasminogen activator (uPA), the glycolipid-anchored cell membrane receptor for the uPA (uPAR) and four serin protease inhibitors (SERPIN), the plasminogen activator inhibitor 1 (PAI-1 or SERPINE1) and 2 (PAI-2 or SERPINB2), the protein C inhibitor (PAI-3 or SERPINA5) and the nexin-1 (SERPINE2) [7-13]. The uPAS is involved in many physiological functions and, along with members of the matrix metalloproteinases (MMPs) family, it has been implicated in cancer invasion and metastatization, in which by degrading ECM and BM allows local diffusion and spread to distant sites of malignant cells [7,8,11,14-17]. A growing number of experimental evidences indicates that the uPAS also affects tumor cell proliferation, migration, adhesion, intravasation and extravasation as well as tumor angiogenesis [8,11,16-21]. The role of uPAS in human cancer progression is further supported by clinical evidences demonstrating that high tissue levels of its components correlate with a poor prognosis in different types of cancer [22-24]. This is particularly evident in breast cancer, in which uPA and PAI-1 have been shown to be among the most potent prognostic factors described to date, with a predictive value stronger than those of patient age, tumor size, estrogen and progesterone receptors, HER-2/neu or p53 expression [17,23-25]. In patients with breast cancer as well as with other types of malignancies, paradoxically, high levels of PAI-1 are also associated with an adverse outcome [10,23-25]. In particular, it has been proposed that high levels of PAI-1 may promote cancer progression in several ways, that is by inhibiting cell adhesions, stimulating cancer cell motility, promoting tumor angiogenesis and preventing an excessive ECM proteolysis by plasmin that could prevent cellular migration [8,10,11,19-21]. In view of their prognostic value, both uPA and PAI-1 are candidate molecular markers for clinical use in patients with breast cancer [8,11]. Moreover, the involvement of uPAS at multiple steps during the neoplastic evolution represents an attractive target for anti-cancer therapy, and a number of studies aimed either to inhibit uPA expression, catalytic activity or to prevent its binding to uPAR have been performed with success on animals, while the results of trials in human cancers are awaited [11,22,26].

To date very few information on the expression of the different uPAS components during the progression of TGCT are available [3,27,28]. In the present study we characterized the expression patterns of uPA, uPAR, PAI-1 and PAI-2 in seminomas tissues in comparison to those present in normal testicular tissues obtained following orchidectomy.

## Methods

### Materials

Trizol^® ^reagent, oligo(dT)_12-18 _primer 0,5 μg/μl and M-MLV reverse transcriptase were purchased from Invitrogen (Carlsbad, CA). All the primers were from Primm (Milano, Italy). Bovine casein, human plasminogen, sodium deoxycholate, aprotinin, leupeptin, phenylmethylsulfonyl fluoride, sodium orthovanadate and sodium pyrophosphate were provided from Sigma Chemical Co. (St. Louis, MO). Bradford protein assay kit and electrophoresis reagents were obtained from Bio-Rad Laboratories (Hercules, CA). Monoclonal antibodies against urokinase plasminogen activator B-chain (uPA) and its receptor (uPAR) were purchased from the American Diagnostica Inc. (Stamford, CT). The monoclonal antibody against actin and the human urokinase native from urine were obtained from Immunological Sciences (Roma, Italy). The anti-mouse peroxidase-conjugated antibody and the SuperSignal chemiluminescent substrate were from Pierce (Rockford, IL). The FastStart DNA Master^PLUS ^SYBR Green I kit was from Roche Applied Sciences (Mannheim, Germany). Perfectprep^® ^Gel Cleanup Kit was obtained from Eppendorf (Hamburg, Germany).

### Normal and tumoral human testicular tissues

Fragments of normal and tumoral testicular tissues were obtained following orchidectomy of patients (age ranging from 24 to 47 years) affected by seminoma treated at the Regina Elena Cancer Institute (Rome). All patients gave their informed consent and the study was approved by the ethical committee of the Regina Elena Cancer Institute. Tissue samples were immediately frozen in liquid nitrogen, stored at -80°C and then used for the preparation of total RNA. In addition, some cDNAs prepared from normal human testis and seminoma tissues were obtained from BioChain Institute, Inc. (Hayward, CA). In total, 14 cases of seminomas and 6 normal human testicular tissues were analyzed (Table 1). Protein extracts were also available from three seminomas and normal matched tissues, *i.e. *normal tissues surrounding the neoplastic lesions obtained from the same patients, and prepared as below described. Finally, 10 paraffin-embedded seminoma tissues, different from those reported in table 1, were available at the Regina Elena Cancer Institute for immunohistochemistry experiments. In the latter, the normal peritumoral tissue was used as control.

**Table 1:** Classification of normal and tumor samples analyzed in the present study. n.a., not available.

Sample ID	Source	Histology	Patient's age	Tumor size (cm)	Stage
**1**	BioChain Inst.	normal	28	-	-
**2**	BioChain Inst.	normal	28	-	-
**3**	Regina Elena Cancer Inst.	normal	25	-	-
**4**	Regina Elena Cancer Inst.	normal	24	-	-
**5**	Regina Elena Cancer Inst.	normal	26	-	-
**6**	Regina Elena Cancer Inst.	normal	41	-	-
**7**	BioChain Inst.	seminoma	41	8	n.a.
**8**	BioChain Inst.	seminoma	24	5	n.a.
**9**	BioChain Inst.	seminoma	44	4	n.a.
**10**	BioChain Inst.	seminoma	30	n.a.	n.a.
**11**	BioChain Inst.	seminoma	34	10	n.a.
**12**	BioChain Inst.	seminoma	43	2	n.a.
**13**	Regina Elena Cancer Inst.	seminoma	24	1	pT2
**14**	Regina Elena Cancer Inst.	seminoma	25	5	pT2
**15**	Regina Elena Cancer Inst.	seminoma	55	6	pT2
**16**	Regina Elena Cancer Inst.	seminoma	26	2	pT2
**17**	Regina Elena Cancer Inst.	seminoma	30	3	pT1
**18**	Regina Elena Cancer Inst.	seminoma	41	4	pT1
**19**	Regina Elena Cancer Inst.	seminoma	27	3	pT2
**20**	Regina Elena Cancer Inst.	seminoma	30	6	pT1

### Extraction and analysis of mRNA

Normal and tumor tissue samples were homogenized in Trizol reagent by the ultra-turrax, and total RNA was extracted with the acid guanidinium thiocyanate-phenol-chloroform method [29]. The purity and integrity of the RNA preparations were checked spectroscopically and by agarose gel electrophoresis before carrying out the analytical procedures. Two-four μg of total RNA were reverse-transcribed and the obtained cDNAs used as a template for the subsequent quantitative PCR amplifications of the different components of the urokinase plasminogen activating system with human β-actin as internal control, using specific primers described in Table 2. Controls for DNA contamination were performed omitting the reverse transcriptase or the RNA during reverse transcription. Quantitative PCR assay was performed with the LightCycler instrument (Roche Diagnostics), employing the FastStart DNA Master SYBR Green I Kit (Roche Applied Sciences). The reactions were set up in a final volume of 20 μl containing heat-activatable *Taq *polymerase, 0.5 μM specific primers and 1.25 ng of template. Following polymerase activation (95°C for 10 min), 40 cycles were run with 10 s denaturation at 95°C, 10 s annealing at optimal temperatures for each primers pair and 25 s extension at 72°C. All samples were processed in triplicate and PCR-grade water was used as negative control. Standard run curves were generated for each gene using 5 serial dilutions of a cDNA mixture expressing all the genes analyzed. The results were analyzed at the end of the run with the LightCycler software, version 1.5 (Roche Diagnostic). In each sample the threshold crossing points (C_t_) of target genes were normalized against that of β-actin, used as reference gene, by the ΔΔC_t _method using the LightCycler relative quantification software 1.0 (Roche Diagnostics). The values of normalized target genes in seminoma samples were divided by the average value of normalized target genes found in 6 normal testicular tissue samples, and reported as fold of variation. All PCR products were analyzed on 2% agarose gel, and to determine the specificities of amplified cDNAs they were recovered from the gel, purified with a gel cleanup kit and subjected to sequencing reactions in presence of fluorescent-labelled nucleotides, then analyzed by ABI Prism 377™ DNA sequencer (Perkin Elmer). All the obtained sequences corresponded to the expected ones (data not shown).

**Table 2:** Primers, genomic positions, size of amplified products and annealing temperatures used in the PCR for the different components of the urokinase plasminogen activating system.

Gene	Primers	Exon	Size (bp)	T_ann._
uPA	forward 5'-GCCATCCCGGACTATACAGA-3' reverse 5'-AGGCCATTCTCTTCCTTGGT-3'	8 10	417	60°C
uPAR	forward 5'-CTGGAGCTGGTGGAGAAAAG-3' reverse 5'-TGTTGCAGCATTTCAGGAAG-3'	3 5	406	60°C
PAI-1	forward 5'-ATACTGAGTTCACCACGCCC-3' reverse 5'-GTGGAGAGGCTCTTGGTCTG-3'	3-4 5-6	320	62°C
PAI-2	forward 5'-GGCCAAGGTGCTTCAGTTTA-3' reverse 5'-GGGATTTTGCCTTTGGTTTG-3'	2-3 5-6	384	62°C
β-Actin	forward 5'-CAAGAGATGGCCACGGCTGCT-3' reverse 5'-TCCTTCTGCATCCTGTCGGCA-3'	3 4	275	62°C

### Western blot

Three normal matched seminoma tissues were homogenized in RIPA buffer (50 mM Tris-HCl pH 7.4, 1% NP-40, 0.5% sodium deoxycholate, 150 mM sodium chloride, 1 mM EDTA, 1 mM sodium fluoride, 1 mM AEBSF, 10 μg/ml aprotinin, 10 μg/ml leupeptin, 1 mM sodium orthovanadate, 10 mM sodium pyrophosphate in ddH_2_O) by ultra-turrax, sonicated and centrifuged at 10.000 rpm for 10 min. The supernatants were then recovered and protein concentrations determined by the Bradford assay [30]. Aliquots of 50 μg of tissue extracts were supplemented with 5× Laemmli buffer (120 mM Tris-HCl, pH 6.8, 2% SDS, 10% Glycerol, 0.01% Bromophenol Blue) and β-mercaptoethanol 0.2% (w/v), heated at 95°C for 5 min., electrophoresed on a 12.5% polyacrylamide gel and transferred onto nitrocellulose membranes using the Biorad Mini Trans-Blot Cell system. The membranes were then washed with TBST (50 mM Tris-HCl, pH 7.5, 150 mM NaCl, 0.05% Tween-20) and saturated with 5% low fat milk in TBST for 2 h at room temperature. Incubations with primary antibodies were performed for the identification of uPA and uPAR in 2.5% low fat milk in TBST at 4°C overnight. The monoclonal antibodies raised against uPA (1:1000) and uPAR (1:250) were detected with anti-mouse (1:1000) horseradish peroxidase-conjugated secondary antibody. Samples loadings in the different western blots were controlled with the monoclonal anti-actin antibody (1:500). The native human urokinase was used as positive control.

### Substrate gel electrophoresis (zymogram)

Fragments of three normal matched seminoma tissue samples were homogenized in RIPA buffer without serine proteases inhibitors by ultra-turrax, sonicated and centrifuged at 10.000 rpm for 10 min. Protein concentrations in the supernatants were determined by the Bradford assay. Fifty μg of tissue extracts were added to 5× zymography sample buffer (0,4 M Tris, pH 6.8, 5% SDS, 20% glycerol, 0.03% bromophenol blue) and electrophoresed on a SDS-polyacrylamide gel containing 0.1% casein plus 12 μg/ml plasminogen as described [31]. After the electrophoretic run, the gel was rinsed once in 50 mM Tris-HCl (pH 7.4) with 2% Triton X-100 and further washed in 50 mM Tris-HCl (pH 7.4). To detect the activity of the plasminogen activators, the gel was incubated at 37°C for 4 h in a buffer containing 50 mM Tris-HCl (pH 7.4) and 0.1% Triton X-100. An identical gel was incubated in the above buffers containing 10 μM aprotinin and 1 mM PMSF in order to assess the enzymes specificity. Finally, the gels were stained with a solution of 0.1% Coomassie brilliant blue in 25% methanol and 7% acetic acid, and destained in the same mixture without dye. Clear zones against the blue background indicated the presence of proteolytic activity. Samples lytic bands were compared with the supernatant of the breast cancer cell line MDA-MB-231, known to secrete both urokinase and tissue type PAs [32]. The molecular weights of each band were evaluated in comparison with a prestained protein ladder using the UVIPRO GEL Documentation System (Eppendorf).

### Immunohistochemical (IHC) analysis

Two μm thick paraffin-embedded sections were treated with trypsin at 37°C for 30' or with citrate buffer (pH 6) for uPA and uPAR antigen retrieval, respectively. Sections were then incubated for 60' at room temperature with anti-uPA antibody or overnight with anti-uPAR antibody. Sections were stained with a streptavidin enhanced immunoperoxidase technique (Supersensitive Multilink, Novocastra, Menarini Florence, Italy), slightly counterstained with Mayer's haematoxylin and mounted in aqueous mounting medium (Glycergel, Dako, Milan, Italy). Immunostained slides were analyzed and scored independently by two investigators (MM, ADB). Concerning uPA and uPAR IHC scoring, seminomas with a missing staining pattern (0) or with a faint immunostaining (1+) for both antigens were classified as negative. Seminomas showing a distinct and intense cytoplasmic immunostaining (2+/3+) were scored as positive regardless of the percentage of stained cells.

### Statistical analysis

The results are expressed as the mean ± SEM of at least three independent experiments. Statistical significance of the expression at mRNA and protein level of the different components of the uPAS in tumor tissues *versus *normal tissues were evaluated by the non parametric Wilcoxon rank sum test using the Epistat computer program. Results from the immunohistochemistry experiments were analyzed by the Fisher's exact test. Correlations between the fold of increase of uPA or uPAR mRNA and tumor size, represented by the major diameter of the lesion, or patient age were evaluated by the non parametric Spearman correlation test using the SPSS software (SPSS Inc., Chicago, Ill.). Results were determined to be significantly different if p values were lower than 0.05.

## Results

**Figure 1:**
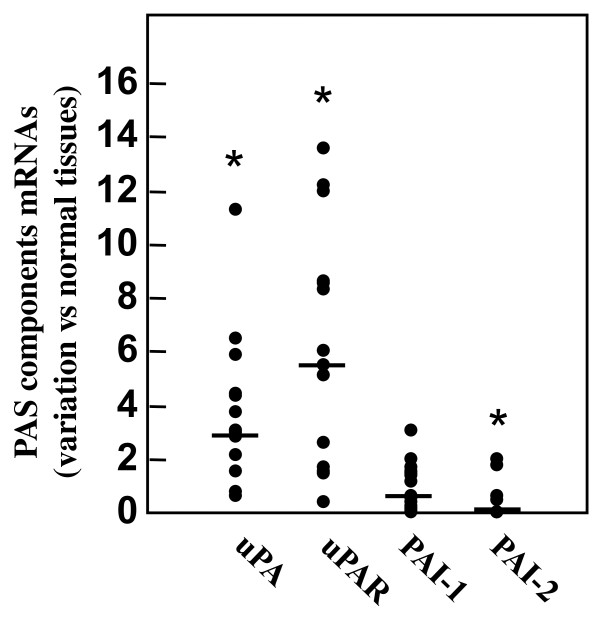
**Messenger RNA levels of the uPAS components in 14 human seminomas analyzed by quantitative RT-PCR**. Fold of variations for uPAS components mRNAs in seminoma tissues have been calculated considering equal to 1 the mean value of uPAS component/β-Actin ratios found in 6 normal testicular tissues. The bars reported in the figure represent the median values. *p < 0.01.

**Figure 2:**
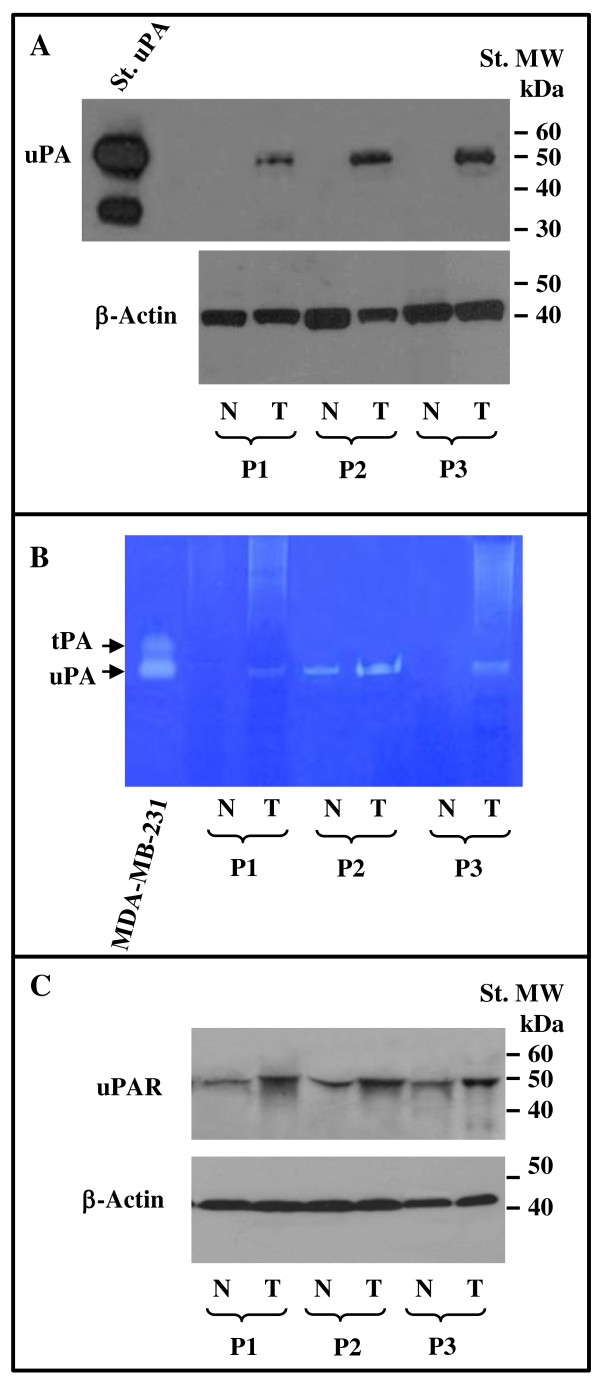
**Western blot analysis of uPA (A) and uPAR (C) and zymographic analysis (B) of 3 normal matched seminoma cancer tissues**. Fifty μg of the different tissue protein extracts were loaded in each lane and subjected to western blot as described in the Methods section, using specific monoclonal antibodies against uPA (panel A), uPAR (panel C) and β-actin as protein loading control. In panel B, is reported the zymographic analysis of the uPA activity in protein tissues extracts of 3 normal matched seminoma cancer tissues. The conditioned medium of human breast carcinoma cell line MDA-MB-231 has been used as positive control for urokinase and tissue type PA activity, as described in the Materials and Methods section. Data reported represents one out of three similar experiments.

We first evaluated the mRNA levels of the different components of the urokinase plasminogen activating system (uPAS) in 14 seminoma tissues in comparison to those observed in 6 normal testicular tissues. Quantitative RT-PCR analysis showed significant (p < 0.01) increases for uPA mRNA by 3.80 ± 0.74 fold, and uPAR mRNA by 6.25 ± 1.18 fold in tumor tissues with respect to normal tissues (figure 1). The level of PAI-1 mRNA was not significantly changed (1.02 ± 0.24 fold), while PAI-2 mRNA level was, with respect to control tissues, significantly reduced in seminomas to 0.34 ± 0.18 fold (p < 0.01) (figure 1). The increased expression of uPA could be confirmed at protein level by means of western blot and zymographic analysis on protein extracts from three available normal matched seminomas. In fact, as reported in figure 2A, western blot experiments showed that uPA protein levels were very low or undetectable in normal tissues and strongly induced in tumor tissues. The enzymatic activity of the plasminogen activators (PAs) present in normal matched tumor tissue protein extracts was evaluated by zymographic analysis, in comparison to that exhibited by the supernatant of the breast cancer cell line MDA-MB-231, known to secrete both urokinase and tissue type PAs [29]. The results reported in figure 2B revealed augmented uPA activity in tumor tissue extracts with respect to the control ones. On the other hand, tPA activity could not be documented either in normal or tumor samples (figure 2B). Incubation of gel with PMSF and aprotinin abrogated all enzyme activities (data not shown). In addition, the increased expression of uPAR could be confirmed at protein level in three normal matched tumor tissue extracts by means of western blot, as reported in figure 2C. Densitometric analysis of the immunoreactive bands showed a significant increase of the uPAR protein in tumor tissues by 1.83 ± 0.15 fold (p < 0.01), with respect to control tissues.

**Figure 3:**
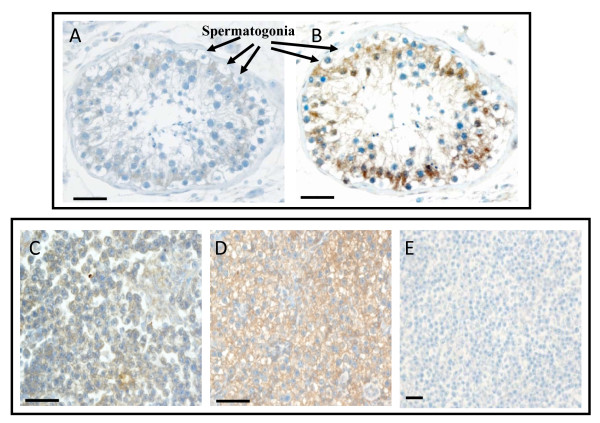
**Immunohistochemistry analysis of uPA and uPAR expression in human testicular germ cell tumor**. Tissue sections from 10 cases of seminomas were incubated with antibodies against human uPA or uPAR and processed as described in the Methods section. **A) **A representative normal testis stained with uPA antibody. **B) **A representative normal testis stained with uPAR antibody. **C) **A representative seminoma stained with uPA antibody. **D) **A representative seminoma stained with uPAR antibody. **E) **Negative control for seminoma tissue obtained omitting the first antibody. Tissue sections were counterstained with Mayer hematoxylin. Scale bar = 30 μm.

IHC staining, performed on 10 human seminomas, showed no uPA reactivity in the autologous normal testis surrounding the tumor (panel A, figure 3), while an intense uPA immunoreactivity was evidenced (score 2+/3+) in 3 out of 10 (30%) samples (p = 0.105) (panel C, figure 3). Staining of uPAR was appreciated in 3 out of 10 normal tissues (30%). In the latter, Sertoli cells appeared to be positive, while the spermatogonia were negative (panel B, figure 3). An intense and homogeneous uPAR immunoreaction (score 2+/3+) was present in 9 (90%) seminomas out of 10 (p < 0.05) (panel D of figure 3). It may be worth mentioning that the single uPAR negative sample showed a strong uPA immunoreactivity. A negative control for seminoma tissue is reported in panel E of figure 3.

**Figure 4:**
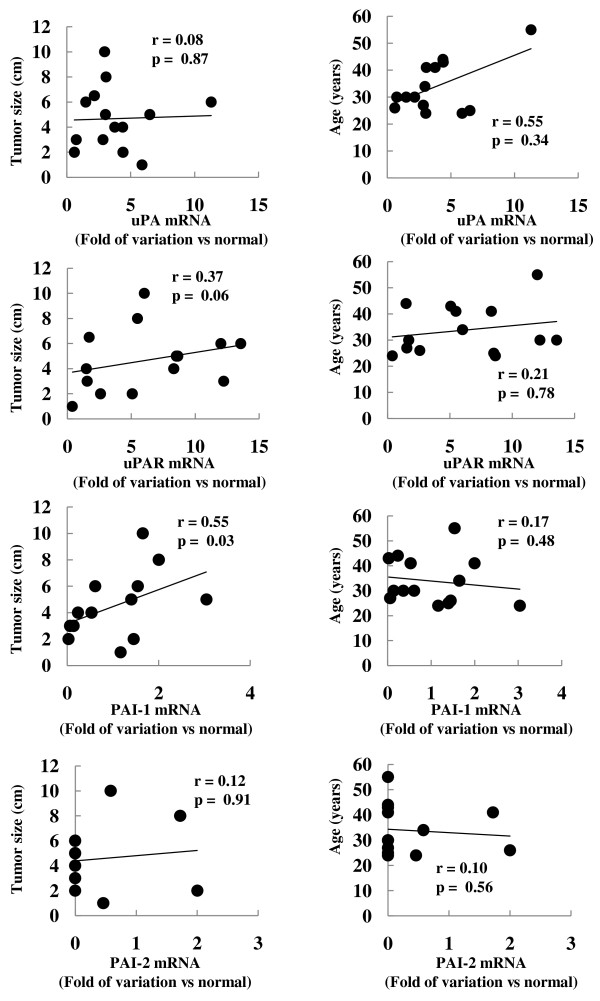
**Correlation analysis between tumor size or patient's age and uPA or uPAR mRNA variations**. Folds of mRNA variations in seminomas *versus *normal tissues were calculated as described in Materials and Methods section. The correlation analysis between uPA or uPAR mRNA variations and tumor size or patient's age were performed by the Spearman correlation test.

We then sought to verify whether uPA, uPAR, PAI-1 or PAI-2 mRNAs expression in tumor tissues correlated with patient's age (n = 14), TNM stage (n = 8, 3 pT1 and 5 pT2) or tumor size (n = 13) (Table 1). As shown in figure 4, among the different uPAS components only PAI-1 significantly correlated with tumor size (p < 0.05), while none of them correlated with patient's age. Finally, none of the uPAS components correlated with TNM stage (data not shown).

## Discussion

Human cancers progression is characterized by malignant cells acquisition of novel functional competences which include self-sufficiency in growth signals, insensitivity to anti-growth signals, evasion of apoptosis, limitless replicative potential, sustained angiogenesis and tissue invasion and metastasis [33]. The aberrant expression of uPAS components in malignant tissues contributes to the acquisition of some of these new cell capabilities. In fact, beside its role in ECM degradation allowing tumor progression and metastasis, extensive experimental evidence demonstrated the capability of the uPAS to affect tumor cell proliferation, adhesion and migration, intravasation and extravasation, growth at the metastatic sites and tumor neoangiogenesis [11,16,17]. The involvement of uPAS in cancer progression and the observation that its inhibition is devoid of toxicity, as demonstrated in uPA or uPAR deficient mice, identifies the uPAS as a suitable target for anti-cancer therapies [11,16,17,26]. Despite that, few information are available on the expression and function of uPAS components in the progression of testicular germ cell tumors (TGCT) [3,27,28,34]. In particular, Okada and colleagues, by means of laser-capture microdissection and a genome-wide cDNA microarray, found the uPA gene (PLAU) among the 347 genes commonly up-regulated in seminoma cells, with respect to normal human testis [28]. In similar studies, the PLAU gene expression was found, with respect to normal testicular tissues, unchanged in the intratubular germ cell neoplasia (IGCN), but significantly augmented in TGCT [3,34]. In the same studies, it was also shown that the expression of the PAI-1 gene did not change significantly in the TGCT [3,34]. These observations are in agreement with the data here reported showing the up-regulation of uPA, but not PAI-1, mRNA in seminomas, with respect to normal testis. The increased expression of uPA was further documented by means of western blot and zymographic analysis of protein extracts from three normal matched tumor tissues. Moreover, we here demonstrated that also the expression of the uPA receptor (uPAR) was significantly increased in seminomas, at both protein and mRNA levels. On the contrary, we found that the expression of PAI-2 gene was significantly reduced in tumor tissues. The latter findings are in partial agreement with the observation of Konaka and colleagues which demonstrated, in a human seminoma xenograft model, the increased expression of uPA, uPAR and PAI-1, and a decreased expression of PAI-2 in testicular xenograft, with respect to subcutaneous xenograft [27]. The expression at the mRNA level of PAI-2 has been associated to a good prognosis in several cancer types such as breast, head and neck, esophageal and pancreatic cancers, and with a poor prognosis in others as endometrial, cervical and colorectal cancer. The latter may be due to the fact that the majority of PAI-2 protein is not secreted but retained in the cells, where it may have a role in protecting the cells from apoptotic stimuli (i.e. TNF-α) [9,10,35,36]. However, the prognostic relevance of PAI-2 in TGCT remains to be investigated.

The immunohistochemical (IHC) analysis revealed, in normal testis surrounding the tumor, that Sertoli cells were weakly positive for uPA and more strongly for uPAR, while spermatogonia were negative for both uPA and uPAR. These observations are in agreement with a previous study reporting the expression of uPA within normal human testis [37]. In the latter, uPA immunoreactivity was detectable in Sertoli cells and in some Leydig cells, but not in spermatogonia [37]. The IHC analysis of tumor tissues confirmed mRNA and protein data obtained by means of RT-PCR and western blot. In fact, we found a statistically significant increase of uPAR protein expression in seminoma with respect to the autologous normal testis surrounding the tumor. Also uPA immunoreactivity was found increased in seminoma tissues with respect to normal testis, but the increase did not reach the statistical significance.

Among the implications of the enhanced uPA expression and activity, strengthened by the concomitant uPAR increase and PAI-2 reduction, is the potential activation by plasmin of latent MMPs, such as proMMP-1, proMMP-2, proMMP-3, proMMP-9, proMMP-10 and proMMP-13. In fact, several MMPs, including MMP-9, show an increased expression in TGCT and, along with plasmin, they might contribute to release or activate ECM-associated mitogenic, motogenic and angiogenic growth factors, including bFGF, VEGF, HGF, IGFs, EGF and TGF-β [3,8,11,16,17,19-21,26,28,38].

Consistent with its role in cancer progression, a strong correlation between the overexpression of one or more uPAS components and the poor clinical outcome in several types of human cancer has been documented [8,11,16,17,39]. In the present study we found that among the uPAS components only PAI-1 significantly correlates with tumor size, while none of them correlates with patient's age or TNM stage. Increased PAI-1 expression has been associated with poor prognosis in several human malignancies, however, since this analysis has been performed on a small number of patients, larger case studies are needed to prove that PAI-1 expression may represent a negative prognostic marker also in human seminoma.

## Conclusions

In conclusion, we demonstrated that testicular germ cell tumors are characterized by increased expression of uPA and uPAR and concomitant reduced expression of PAI-2. This could be of relevance in seminoma progression promoting local diffusion and spread to distant sites.

## List of abbreviations

uPAS: urokinase plasminogen activating system; uPA: urokinase plasminogen activator; uPAR: urokinase plasminogen activator receptor; PLAU: plasminogen activator, urokinase; PAI: plasminogen activator inhibitor; SERPIN: serine protease inhibitor; TGCT: testicular germ cell tumor; IGCN: intratubular germ cell neoplasia; ECM: extracellular matrix; BM: basement membrane; MMP matrix metalloprotease; IHC: immunohistochemistry; T: the TNM staging system is based on the extent of the tumor; N: spread to lymph nodes; and M: metastasis; bFGF: basic fibroblast growth factor; VEGF: vascular endothelial growth factor; HGF: hepatocyte growth factor; IGFs: insulin-like growth factors; EGF: epidermal growth factor; TGF-β: transforming growth factor.

## Competing interests

The authors declare that they have no competing interests.

## Authors' contributions

SU, PG, EDA MDA contribute to conception and design of the experiments described and drafted the manuscript; SU and BV also carried out the RNA extraction and real-time PCR to evaluate the expression of the different components of the plasminogen activating system; EB carried out the western blots and the zymographic analysis; MM, SS and ADB provided the histopathological classification of tumor samples, performed and analyzed the results of the immunohistochemistry experiments and drafted the manuscript; SS, PG and MDA contribute to the statistical analysis of the experimental data. All authors read and approved the final manuscript.

## Pre-publication history

The pre-publication history for this paper can be accessed here:

http://www.biomedcentral.com/1471-2407/10/151/prepub
